# Präklinische Versorgung schwer verletzter Kinder in Deutschland: Epidemiologie, Maßnahmen und strukturelle Perspektiven

**DOI:** 10.1007/s00101-025-01624-4

**Published:** 2026-01-09

**Authors:** Lennart Burger, Sebastian Rehberg, Karl-Christian Thies

**Affiliations:** 1 Medizinische Fakultät, Universität Bielefeld, Bielefeld, Deutschland; 2 Medizinische Fakultät, Heinrich-Heine-Universität Düsseldorf, Düsseldorf, Deutschland; 3 Medizinische Fakultät und Universitätsklinikum OWL, Ev. Klinikum Bethel, Universitätsklinik für Anästhesiologie, Intensivmedizin, Notfallmedizin, Transfusionsmedizin und Schmerztherapie, Universität Bielefeld, Bielefeld, Deutschland

**Keywords:** Pädiatrisches Trauma, Schädel-Hirn-Trauma, Jugendliche, Rettungsdienst, Luftrettung, Pediatric trauma, Traumatic Brain Injury, Adolescent, Emergency medical services, Air ambulances

## Abstract

**Hintergrund:**

Schwer verletzte Kinder machen nur einen kleinen Anteil der Einsätze im Rettungsdienst aus, stellen die Einsatzteams aber aufgrund altersabhängiger Anatomie und Physiologie, komplexer Verletzungsmuster und hoher emotionaler Belastung vor große Herausforderungen. Diese narrative Übersichtsarbeit fasst die Evidenz zu Epidemiologie und präklinischer Versorgung pädiatrischer Traumapatienten im deutschen Rettungsdienst zusammen und ordnet die Befunde in einen internationalen Kontext ein.

**Material und Methoden:**

Es wurde eine PubMed-Suche mit den Begriffen „children“, „trauma“ und „prehospital“ durchgeführt. Eingeschlossen wurden Studien zur präklinischen Versorgung verletzter Kinder und Jugendlicher; von 421 Treffern wurden 20 Arbeiten ausgewählt und durch Rückwärtssuche ergänzt, mit Fokus auf Daten aus Deutschland und vergleichbaren Hochlohnländern.

**Ergebnisse:**

Pädiatrische Notfälle machen etwa 5 % der bodengebundenen und 6–13 % der luftgebundenen Einsätze aus; schwer verletzte Kinder (z. B. NACA IV–VII) stellen nur einen kleinen Bruchteil dar und machen das pädiatrische Polytrauma zu einem klassischen „Low-frequency-high-impact“-Szenario. Der Anteil traumatischer Notfälle steigt mit dem Alter und ist im Jugendalter am höchsten; Jungen sind in allen Kollektiven überrepräsentiert. Stürze und Verkehrsunfälle dominieren, überwiegend als stumpfe Traumata mit vorwiegend Kopf- und Extremitätenverletzungen. Ein Polytrauma wird nur bei rund 5 % der verletzten Kinder beschrieben und betrifft v. a. ältere Kinder und Jugendliche. Sauerstoffgabe, venöse Zugänge, Immobilisation und Analgesie gehören zu den häufigen Maßnahmen, während Intubation, intraossäre Zugänge, Thoraxdrainagen und andere invasive Prozeduren selten bleiben. Mehrere Studien belegen, dass Luftrettung sowie die primäre Aufnahme in ein (pädiatrisches) Traumazentrum mit einer verbesserten Überlebensrate assoziiert sind.

**Schlussfolgerung:**

Pädiatrische Traumapatienten bilden eine kleine, aber risikobehaftete Patientengruppe, bei der invasive Prozeduren unter Zeitdruck und emotionaler Belastung durchgeführt werden müssen. Die Versorgung komplexer Fälle durch spezialisierte HEMS-Teams und in klar ausgewiesenen (pädiatrischen) Traumazentren, eine gestärkte notfallsanitäterbasierte Versorgung, simulationsbasiertes Training und eine standardisierte prä- und innerklinisches Outcome-orientierte Datenerfassung sowie der weitere Ausbau regional koordinierter Traumanetzwerke sind zentrale Schritte zur Verbesserung der präklinischen Versorgung verletzter Kinder.

## Abstract (en)

**Background:**

Severely injured children account for only a small proportion of emergency medical service (EMS) missions but pose major challenges to the emergency teams due to age-related anatomy and physiology, complex injury patterns and high emotional stress. This narrative review summarizes the current evidence on the epidemiology and prehospital management of pediatric trauma patients in the German EMS system and places these findings in an international context.

**Material and methods:**

A PubMed search was carried out using the terms “children”, “trauma” and “prehospital”. Studies reporting the prehospital management of injured children and adolescents were included. Out of 421 records 20 studies were selected and complemented by backward citation searches, with a focus on data from Germany and comparable high-income countries.

**Results:**

Pediatric emergencies account for approximately 5% of ground EMS (GEMS) and 6–13% of helicopter EMS (HEMS) missions; severely injured children (e.g. NACA IV–VII) constitute only a small fraction, making pediatric polytrauma a classical low-frequency, high-impact scenario. The proportion of traumatic emergencies increases with age and peaks during adolescence; boys are overrepresented in all cohorts. Falls and traffic incidents dominate, predominantly resulting in blunt trauma with injuries mainly to the head and extremities. Polytrauma is reported in only around 5% of injured children and primarily affects older children and adolescents. Oxygen administration, intravenous access, immobilization and analgesia are frequent measures, whereas intubation, intraosseous access, chest drainage and other invasive procedures are rare. Several studies indicate an association between HEMS or direct admission to a (pediatric) major trauma center and improved survival.

**Conclusion:**

Pediatric trauma patients represent a small but high-risk group in whom invasive procedures must be performed under time pressure and emotional stress. The management of complex cases by specialized HEMS teams and clearly designated (pediatric) major trauma centers, the strengthening of paramedic-based care, simulation-based training, standardized prehospital and in-hospital outcome-oriented data collection and the further expansion of regionally coordinated trauma networks are key steps towards improving prehospital care for injured children.

## Einleitung

Verletzungen zählen weltweit zu den größten Gesundheitsrisiken im Kindes- und Jugendalter [73]. Trotz rückläufiger Gesamtsterblichkeit bleibt das Trauma in Europa eine der häufigsten Todesursachen in dieser Altersgruppe [66]. Viele Überlebende tragen langfristige körperliche und psychische Folgen mit entsprechenden Belastungen für Familien, Gesundheitssysteme und Gesellschaft [38, 58]. Neben Prävention und Verkehrssicherheit ist insbesondere die Qualität der präklinischen Versorgung entscheidend für das funktionelle Outcome [38].

Schwer verletzte Kinder sind im Rettungsdienst selten, zugleich aber besonders anspruchsvoll. Altersabhängige anatomische und physiologische Besonderheiten und die hohe emotionale Belastung für das Einsatzteam erfordern spezifisches Fachwissen und Handlungssicherheit. Geringe Fallzahlen, begrenzte praktische Erfahrung und unzureichende Aus- und Weiterbildungsmöglichkeiten erschweren dies zusätzlich; komplexe Maßnahmen werden nur selten durchgeführt [49].

Die deutsche Traumaversorgung ist zunehmend durch Spezialisierung und Zentralisierung geprägt. Während die Versorgung in Traumazentren die Ergebnisqualität verbessert [5], führt der fortschreitende Rückbau pädiatrischer Notfall- und stationärer Versorgungsstrukturen zu längeren Transportwegen und einer wachsenden Herausforderung für den Rettungsdienst [2, 71]. Belastbare nationale Daten zur präklinischen Versorgung verletzter Kinder liegen bislang nur eingeschränkt vor. Das TraumaRegister DGU® liefert wichtige Informationen zur innerklinischen Behandlung Schwerverletzter, erfasst jedoch die präklinische Phase nur retrospektiv über die aufnehmenden Kliniken. Im Jahresbericht 2023 wurden über 38.500 Schwerverletzte dokumentiert, davon rund 10 % Kinder und Jugendliche [5, 72].

Internationale Register wie das britische National Major Trauma Registry (NMTR) oder die US-amerikanische National Trauma Data Bank (NTDB) liefern umfangreiche Daten zu innerklinischen Abläufen und Outcomes, teils auch mit präklinischen Parametern. Ihre Ergebnisse bieten Einblicke in alternative Versorgungsstrukturen, Ausbildungsstrategien und klinische Protokolle.

Ziel unserer narrativen Übersichtsarbeit ist es, epidemiologische Merkmale, Verletzungsmuster und präklinische Interventionen bei pädiatrischen Traumapatienten im Rettungsdienst darzustellen, die deutsche Datenlage im internationalen Kontext einzuordnen und Ansatzpunkte zur Weiterentwicklung der präklinischen Versorgung von Kindern abzuleiten.

## Methodik

Diese narrative Übersichtsarbeit basiert auf einer PubMed-Recherche mit den Suchbegriffen „children“, „trauma“ und „prehospital“ (Suchdatum: 04.02.2025; Trefferzahl *n* = 421). Eingeschlossen wurden englisch- und deutschsprachige Originalarbeiten, Reviews und Metaanalysen (Publikationszeitraum 1999–2025), die Daten zur präklinischen Versorgung verletzter Kinder und Jugendlicher enthielten. Arbeiten ohne klaren Bezug zur präklinischen Traumaversorgung wurden ausgeschlossen.

Aus den identifizierten Publikationen wurden jene mit direktem Bezug zur Epidemiologie, zu Unfallmechanismen und zur klinischen Präsentation pädiatrischer Traumapatienten im Rettungsdienst ausgewählt (*n* = 20). Besonderes Augenmerk lag auf Studien mit Primärdaten aus dem deutschen Rettungsdienst (u. a. Luftrettung, bodengebundene Systeme). Die Schlüsselpublikationen mit Daten aus dem deutschen Rettungsdienst sind in Tab. 1 zusammengefasst.

**Tab. 1:** Kerndaten aus Schlüsselpublikationen der vergangenen 25 Jahre zu schwer erkrankten und verletzten Kindern im deutschen boden- und luftgebundenen Rettungsdienst. In allen Analysen wurden jeweils nur Einsätze ärztlich besetzter Rettungsmittel, also Notarzteinsatzfahrzeuge (Ground Emergency Medical Services, *GEMS*) und Rettungshubschrauber (Helicopter Emergency Medical Services, *HEMS*), ausgewertet.

Autoren (Jahr)/Beobachtungszeitraum	Setting	Alterskohorte	Gesamteinsatzzahl (absolut)	Anteil der Kindereinsätze (%)	Davon Trauma (%)	Davon schwer verletzte Kinder (NACA IV–VII) (%)
Albrech et al. (2000); 1994–1996 [1]	HEMS – ‚Christoph 16‘ Saarbrücken	≤ 14 Jahre	HEMS: 3.305	HEMS: 11,1	HEMS: 58	HEMS: 30
Schlechtriemen et al. (2006); 2001–2003 [64]	HEMS – 27 Standorte und GEMS – 15 Standorte	< 18 Jahre	GEMS: 68.158; HEMS: 71.726	GEMS: 6,4; HEMS: 12,9	GEMS:35,6; HEMS: 59,9 (0,5 vs. 5 Polytrauma)	GEMS: 15; HEMS: 30,5 *
Eich et al. (2009); 1998–2006 [15]	HEMS und GEMS – Standort am Universitätsklinikum Göttingen	≤ 14 Jahre	36.240 (GEMS + HEMS)	Gesamt: 6,3; GEMS: 5,2; HEMS: 8,5	Gesamt: 32,4; GEMS: 21,8; HEMS: 61,1	21,4 (Anteile für GEMS und HEMS nicht getrennt angegeben)
Helm et al. (2010); 2004–2007 [27]	HEMS – ‚Christoph 22‘ Ulm	< 18 Jahre	HEMS: 5.826	HEMS: 11 (13,5 der Primäreinsätze)	HEMS: 57,9 (≥ 6 Jahre > 70)	HEMS: 59,3*
Bernhard et al. (2011); 2005–2006 [3]	HEMS und GEMS – 3 Standorte	< 18 Jahre	GEMS: 17.547; HEMS: 2.929	GEMS: 4,3; HEMS: 11,5	Gesamt: 43,2; GEMS: 36,8; HEMS: 57,6	GEMS: 20,4; HEMS: 55,5*
Mockler et al. (2023); 2014–2018 [48]	HEMS (DRF Luftrettung, 31 Standorte)	≤ 10 Jahre	HEMS: 127.964	HEMS: 6,2	HEMS: 56,5 (4,9 Polytrauma)	HEMS: 24,2

Bei den mit * markierten Angaben zum NACA-Score wurde in den Arbeiten nicht zwischen erkrankten und verletzten Kindern differenziert

Abkürzungen s. Abkürzungsverzeichnis

Die ausgewählten Arbeiten wurden hinsichtlich Studiendesign, Kollektivgröße, Datengrundlage und Übertragbarkeit auf das deutsche Versorgungssystem qualitativ bewertet und thematisch geordnet (Prävalenz, Alters- und Geschlechtsverteilung, Verletzungsmuster, präklinische Maßnahmen). Ergänzend erfolgte eine Rückwärtssuche über die Literaturverzeichnisse der eingeschlossenen Studien, um relevante zusätzliche Datenquellen zu identifizieren.

## Ergebnisse

### Prävalenz

Die Prävalenz kindlicher Notfälle im Rettungsdienst liegt international – je nach Altersdefinition und Systemstruktur – meist bei etwa 5–10 % aller Einsätze, in einzelnen Studien bei bis zu rund 25 % [3, 13, 15, 22, 27, 28, 40, 46, 48, 51, 56, 64, 65]. In deutschen Untersuchungen entfielen im bodengebundenen Rettungsdienst (GEMS) etwa 5 % der Einsätze auf Kinder [3, 15, 64]; in der Luftrettung lag der Anteil mit 6–13 % etwas höher, was u. a. an unterschiedlichen Altersgrenzen liegt [1, 3, 15, 27, 48, 64].

Bei unter 18-Jährigen waren in der Luftrettung (HEMS) rund 60 % der Primäreinsätze traumabedingt; etwa ein Drittel dieser Kinder wurde als schwer verletzt (NACA IV–VII) eingestuft und machte damit nur etwa 2–3 % des gesamten Einsatzaufkommens aus [1, 3, 15, 27, 48, 64]. Im bodengebundenen Rettungsdienst (GEMS) lag der Traumaanteil bei Kindern bei ungefähr 35 % [3, 64]. Eine entsprechende NACA-Auswertung explizit für verletzte Kinder im deutschen GEMS ist nach Kenntnis der Autoren jedoch nicht publiziert, sodass hier – anders als in der Luftrettung – keine belastbaren Angaben zum Schweregrad vorliegen. Internationale Daten zeigen ein Bild mit kleinen Kinderkollektiven und hohem Schweregrad in selektierten HEMS-Systemen: In Österreich und Dänemark wurden etwa 30 % der pädiatrischen Patienten als NACA IV–VII klassifiziert [54, 65]; niederländische Studien berichteten in HEMS-Kollektiven von hohen Anteilen schwer kranker Kinder und Mortalitätsraten um 10–17 % [22, 56]. Eine große US-Analyse mit über 127.000 pädiatrischen Traumapatienten fand bei ISS ≥ 9 einen Überlebensvorteil nach HEMS-Transport, gleichzeitig waren etwa 40 % der Transportierten nur leicht verletzt (ISS ≤ 8), was auf relevante Übertriage hinweist [18].

Langzeitdaten zur Inzidenz schwerer Verletzungen bei Kindern sind heterogen. Einige Kohorten berichteten stabile oder steigende Fallzahlen [11, 62], während eine norwegische Untersuchung einen Rückgang der traumabedingten Mortalität um 57 % innerhalb von 2 Jahrzehnten zeigte [30]. Insgesamt wird in Europa ein rückläufiger Trend beschrieben, der in Ländern mit mittlerem und niedrigem Einkommen weniger ausgeprägt ist [66]. Für Deutschland ist ein deutlicher Rückgang verkehrsbedingter Verletzungen dokumentiert: Zwischen 1978 und 2021 sank die Zahl der im Straßenverkehr verunglückten Kinder von 468/100.000 Einwohner auf 194/100.000 Einwohner [67].

### Alters- und Geschlechtsabhängigkeit

**Abb. 1:**
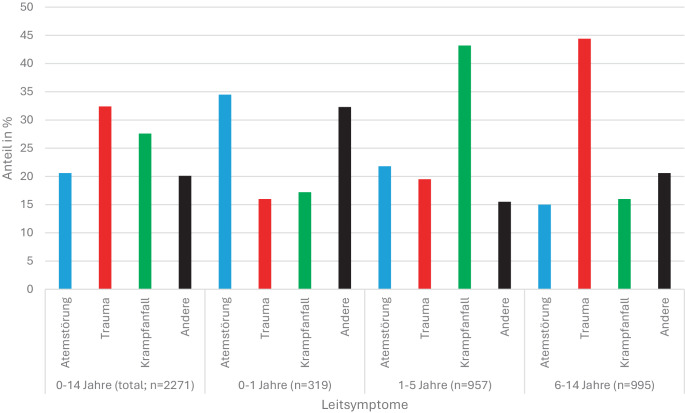
Leitsymptome bei prähospitalen Kindernotfällen (0 bis 14 Jahre) (GEMS und HEMS). Dargestellt sind die Anteile (in %) von Atemstörungen (*blau*), Traumata (*rot*), Krampfanfällen (*grün*) und anderen (*schwarz*) im Gesamtkollektiv und in der Gruppe der Säuglinge (< 1 J.), Kleinkinder (1–5 J.) und Schulkinder (6–14 J.). Die Kategorie „Andere“ umfasst seltene bzw. nicht näher spezifizierte Leitsymptome. Modifiziert nach Eich et al. [15]

Mit zunehmendem Alter steigt der Anteil unfallbedingter Notfälle deutlich an. In einer deutschen monozentrischen Analyse von Helm et al. dominierten bei Säuglingen internistische und neurologische Notfälle. Der Traumaanteil lag bei 15,8 %; dieser stieg bei Kleinkindern auf 43,2 %, bei Schulkindern auf 64 % und lag bei 10- bis 18-Jährigen über 70 % [27]. Eich et al. beschrieben ein ähnliches Muster für Boden- und Hubschraubernotarzteinsätze: Atemstörungen standen im Säuglingsalter im Vordergrund, Krampfanfälle bei Kleinkindern, Traumata bei Schulkindern (Abb. 1; [15]).

Internationale Daten bestätigen die altersabhängige Zunahme traumatischer Verletzungen; schwere Traumata häufen sich besonders im Jugendalter [11, 40, 52, 65]. Entsprechend weisen Daten aus dem TraumaRegister DGU® und der BURDEN-Studie auf eine erhöhte traumabedingte Mortalität bei Jugendlichen im Vergleich zu jüngeren Kindern hin [10, 58].

Nahezu alle Quellen zeigen eine Dominanz männlicher Patienten. Nach Daten des Statistischen Bundesamts von 2021 verunglückten Jungen im Straßenverkehr häufiger als Mädchen und hatten ein höheres Risiko, an Unfallfolgen zu sterben [67]. Kahl et al. fanden steigende Unfallraten vom Kleinkind- zum Jugendalter bei Jungen, während die Raten bei Mädchen weitgehend konstant blieben; zudem traten Gewaltverletzungen etwas häufiger bei Jungen auf [31]. Die epidemiologische Analyse von Elsässer bestätigte höhere Verletzungs- und Sterberisiken sowie höhere Hospitalisationsraten für Jungen (relatives Risiko je nach Altersgruppe etwa 1,2- bis 1,9fach erhöht) [17]. Im TraumaRegister DGU® zeigte sich bei 517 schwer verletzten Kindern (0 bis 15 Jahre, ISS ≥ 16) eine männliche Dominanz von rund 60 %, ohne signifikante Geschlechtsunterschiede in Schweregrad oder Mortalität [10].

### Nichtakzidentielle Verletzungen

Nichtakzidentelle Gewalteinwirkungen sind selten, jedoch klinisch hochrelevant. Eine systematische präklinische Erfassung fehlt, doch zeigen verfügbare Daten, dass Rettungsdienstpersonal regelmäßig mit Verdachtsfällen konfrontiert ist und häufig Unsicherheiten bestehen [19, 25].

Laut *Polizeiliche Kriminalstatistik 2024* wurden 3.609 Fälle körperlicher Misshandlung von Schutzbefohlenen registriert, insgesamt 4.573 betroffene Kinder; die Inzidenz lag bei 40,9/100.000 Kindern unter 14 Jahren [8]. Aufgrund nichtangezeigter Fälle ist von einer hohen Dunkelziffer auszugehen; Schätzungen zufolge erfahren 5,5–12,7 % aller Minderjährigen körperliche Gewalt durch Eltern, entsprechend 700.000 bis 1,7 Mio. Kindern und Jugendlichen [4].

Die Diagnostik bleibt herausfordernd: In Studien zu misshandlungsbedingtem Schädel-Hirn-Trauma fanden sich in 79 % bereits zuvor dokumentierte Hinweise, dennoch wurde die Diagnose in 62 % der Fälle initial verpasst [41]. Rechtsmedizinische und internationale Analysen bestätigen, dass insbesondere bei Säuglingen und Kleinkindern schwere Misshandlungen häufig nicht erkannt werden [19, 20].

### Unfallmechanismen

**Abb. 2:**
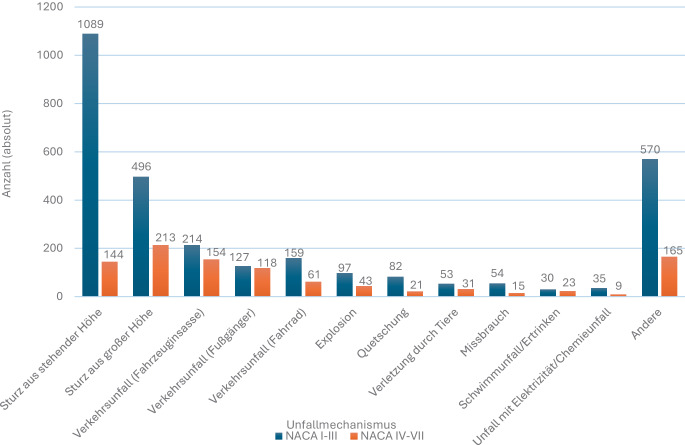
Unfallmechanismen bei durch HEMS versorgten Kindern in der Arbeit von Mockler et al. Dargestellt sind die absoluten Fallzahlen für die verschiedenen Unfallmechanismen, getrennt nach Schweregrad der Verletzung (NACA I–III vs. NACA IV–VII). Die Kategorie „Andere“ umfasst seltene bzw. nicht näher spezifizierte Mechanismen. Modifiziert nach Mockler et al. [48]

In der Promotionsschrift von Mockler waren Stürze (43 %) und Verkehrsunfälle (18,5 %) die häufigsten Unfallmechanismen bei Kindern (Abb. 2); thermische Verletzungen betrafen überwiegend Kinder unter 4 Jahren [49]. Hochenergetische Stürze sowie PKW- und Fußgängerunfälle fanden sich v. a. in den Schweregraden NACA IV–VII, das Kollektiv wurde klar von stumpfen Traumata dominiert, penetrierende Verletzungen traten nur vereinzelt auf [49]. Albrech et al. beschrieben in einer älteren Luftrettungsstudie ein ähnliches Muster mit überwiegend Verkehrsunfällen und Stürzen [1]; weitere deutsche Arbeiten aus Luft- und Bodenrettung bestätigten diese Dominanz [3, 5, 15, 27, 64].

Nach Angaben des Statistischen Bundesamts wurden 2024 rund 27.260 Kinder unter 15 Jahren im Straßenverkehr verletzt. Häufig betroffen waren Schulkinder auf dem Weg zur Schule; sie verunglückten v. a. als PKW-Insassen oder Fahrradfahrer, während Kinder unter 6 Jahren überwiegend als Mitfahrer im Auto beteiligt waren [68].

Internationale Studien berichteten vergleichbare Muster: In einer niederländischen Luftrettungskohorte dominierten stumpfe, unbeabsichtigte Mechanismen (Stürze, Verkehrs- und Freizeitunfälle) bei gleichzeitig hoher 24-h-Mortalität von 13 % [22]. Insgesamt lag in nahezu allen Studien der Anteil stumpfer Verletzungen bei über 85 %, penetrierende Traumata blieben selten und meist unter 10 % [40, 62]. Lebensbedrohliche Blutungen waren überwiegend die Folge stumpfer Traumata; etwa zwei Drittel dieser Fälle wurden darauf zurückgeführt [43].

### Klinische Präsentation und Notfallversorgung

In Mocklers Kollektiv wurde der Zustand von 24 % der verletzten Kinder als kritisch bis lebensbedrohlich (NACA IV–VII) eingestuft, Eich et al. berichteten einen Anteil von etwa 21 % schwer verletzter Kinder [15, 49]. In anderen deutschen Arbeiten wurden ebenfalls hohe Schweregrade dokumentiert: Helm et al. [27] zeigten 59,3 % NACA IV–VII, Bernhard et al. [3] berichteten hingegen 31,2 % NACA IV–VII in einem gemischten prähospitalen Kollektiv. Allerdings differenzierten beide Arbeiten nicht konsequent zwischen Trauma und anderen medizinischen Notfällen.

### Ergänzende nationale und internationale Daten

Eine Untersuchung des Universitätsklinikums Köln zu 373 über den Schockraum aufgenommenen Kindern (99,2 % traumatisiert) zeigte, dass trotz Schockraumzuweisung 44,2 % der Kinder am selben Tag entlassen wurden; 31,1 % wurden auf einer Normalstation, 8,8 % auf der Intensivstation aufgenommen, 8,0 % operiert; die Mortalität lag bei 0,5 % [21]. Dies stützt die Annahme, dass ein erheblicher Teil der pädiatrischen Schockraumpatienten nicht schwer verletzt ist.

Internationale Arbeiten zeigten ein ähnliches Bild mit deutlicher Spannweite: In Dänemark erforderten 50 % der pädiatrischen Notarzteinsätze keine akute Intervention [46], in einer schwedischen Studie verblieben bis zu 25 % der verletzten Kinder nach der Untersuchung am Einsatzort [40]. Demgegenüber wiesen Kinder mit schwerem Trauma in einer spanischen Kohorte in über der Hälfte der Fälle Polytraumata mit ≥ 3 verletzten Körperregionen auf [11]; eine norwegische Analyse zeigte einen altersabhängigen Anstieg schwerer Verletzungen (ISS > 15) von 11 % bei 0‑ bis 4‑Jährigen auf 19 % bei 15- bis 17-Jährigen [52].

### Konkrete klinische Muster

#### Verletzte Körperregionen

**Abb. 3:**
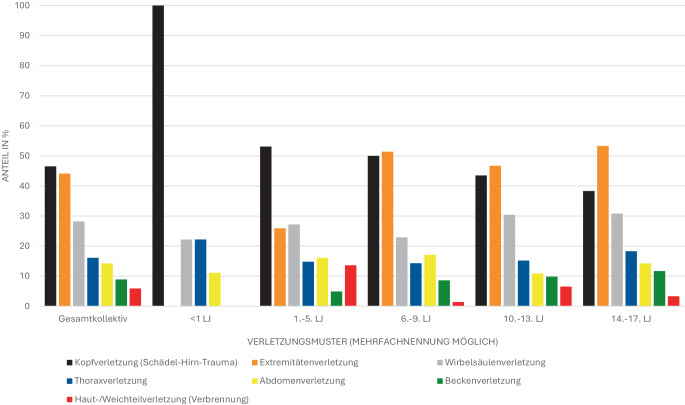
Verletzungsmuster bei pädiatrischen Traumapatienten (0 bis 17 Jahre) in der Arbeit von Helm et al. Dargestellt sind die Anteile (in %) einzelner Verletzungsmuster im pädiatrischen Gesamtkollektiv sowie für einzelne Altersgruppen. Modifiziert nach Helm et al. [27]

Mockler et al. dokumentierten überwiegend Einzelverletzungen; Mehrfachverletzungen traten in 13 %, Polytraumata in rund 5 % der Fälle auf [48]. Eine vergleichbare Polytraumarate beschrieben Schlechtriemen et al. für die Luftrettung [64]. Häufig waren Schädel-Hirn-Traumata (SHT) und Extremitätenverletzungen, während Thorax- und Abdominaltraumata seltener, aber überproportional mit Polytrauma und erhöhter Mortalität verknüpft waren [1, 27, 28, 48, 64]. Mit zunehmendem Alter nahmen SHT ab, während Wirbelsäulen- und Extremitätentraumata häufiger auftraten (Abb. 3; [27, 64]).

#### Neurologische Auffälligkeiten

Mehrere Arbeiten zeigten hohe Anteile neurologisch beeinträchtigter Kinder. Helm et al. beobachteten in einem gemischten pädiatrischen Kollektiv eine GCS-< 9-Rate von 16 % [27], Mockler dokumentierte bei vital bedrohten Kindern 28 % mit GCS < 9 [49]. In schwer verletzten Traumakollektiven lagen die Anteile noch höher: Bläsius et al. beschrieben im TraumaRegister DGU® bei schwer verletzten HEMS-Patienten eine GCS-< 9-Rate von 33 % [5], Helm et al. und Schmidt et al. berichteten insgesamt 32–49 % mit GCS 3–8 [28, 63]. Bernhard et al. identifizierten im Luftrettungsdienst einen hohen Anteil verletzter und erkrankter Kinder mit Bewusstseinsstörung (59,6 %) [3].

#### Atmung und Oxygenierung

Hypoxien waren relativ selten: Mockler dokumentierte eine S_p_O_2_ < 90 % in 3,9 % der Fälle [49]. Atemwegssicherung und Sauerstoffgabe gehörten zu den häufigen Maßnahmen: Helm et al. berichteten Intubationsraten bis 20 % und eine Sauerstoffapplikation bei 76 % der Kinder [27]. In Mocklers Gesamtkollektiv wurden 6,6 % aller Kinder, von den verletzten Kindern etwa 7 % intubiert; Säuglinge wurden häufiger aus medizinischen Gründen, ältere Kinder überwiegend traumaassoziiert intubiert [49]. Hochgerechnet entspricht dies etwa einer pädiatrischen Intubation bei Patienten ≤ 10 Jahren alle 6 Jahre/Notarzt und einer Säuglingsintubation nur etwa alle 46 Jahre.

#### Kreislaufinstabilität

In der TR-DGU®-Analyse von Bläsius et al. wiesen etwa 15 % der schwer verletzten Kinder präklinisch systolische Blutdruckwerte < 90 mm Hg auf [5]. Diese Hypotonien traten überwiegend bei Patienten mit Polytrauma und hohen ISS-Werten sowie mit schweren Thorax- und Abdominalverletzungen auf. Insgesamt scheinen manifeste Schockzustände in den präklinischen Dokumentationen zwar relativ selten erfasst zu werden, betreffen dann aber v. a. schwer verletzte Subgruppen.

#### Extremitätentrauma/Analgesiebedarf

Extremitätenverletzungen gehören in allen ausgewerteten Studien zu den häufigsten Verletzungsmustern. Mockler et al. dokumentierten bei verletzten Kindern eine regelmäßige Analgetikagabe mit Opioiden (26 %) und Nichtopioiden (22,6 %) sowie hohe Raten an Immobilisationsmaßnahmen [48, 49]. In einer aktuellen Analyse von Daten der DRF Luftrettung hatten 38,4 % der pädiatrischen Traumapatienten bei Erstkontakt moderate oder starke Schmerzen (NRS > 4); bis zur Klinikübergabe sank dieser Anteil auf 4,9 % (≈ 95,1 % mit NRS ≤ 4); es kamen hauptsächlich Fentanyl und Ketamin zur Anwendung [16]. In der Studie von Helm et al. wurden bei rund 87 % der traumatisierten Kinder Extremitäten immobilisiert oder reponiert und zusätzlich Zervikalstützen oder Vakuummatratzen angelegt [27].

#### Gefäßzugang

Bei den meisten verletzten Kindern gelingt präklinisch ein venöser Zugang. Mockler berichtete bei traumatisierten Kindern über eine i.v.-Zugangsrate von 62,7 % und eine intraossäre Rate von 3,5 %; in der Subgruppe vital bedrohter Kinder lag die kombinierte i.v.-i.o.-Rate bei 92,8 % [49]. Ähnliche i.v.-Raten um 80 % fanden sich bei Albrech et al. und Helm et al.; intraossäre Zugänge blieben im Gesamtkollektiv selten und betrafen überwiegend Kinder unter 6 Jahren [1, 27]. Etwa die Hälfte der Kinder erhielt eine moderate Volumentherapie zwischen 50 und 500 ml; Infusionsmengen > 1000 ml waren selten und fast ausschließlich bei traumatisierten Kindern erforderlich [49].

#### Thoraxdrainagen und weitere invasive Maßnahmen

Thoraxdrainagen wurden nur in Einzelfällen eingesetzt: Helm et al. berichteten im Luftrettungsdienst bei traumatisierten Kindern eine Rate von 2,2 % [27]; Mockler beschrieb ebenfalls nur Einzelfälle [49]. Weitere invasive Maßnahmen wie Thorakotomien oder chirurgische Atemwegssicherungen wurden in den meisten Studien gar nicht oder nur sporadisch erwähnt und lassen sich auf Basis der verfügbaren Daten nicht belastbar quantifizieren.

### Kinder vs. Erwachsene

**Abb. 4:**
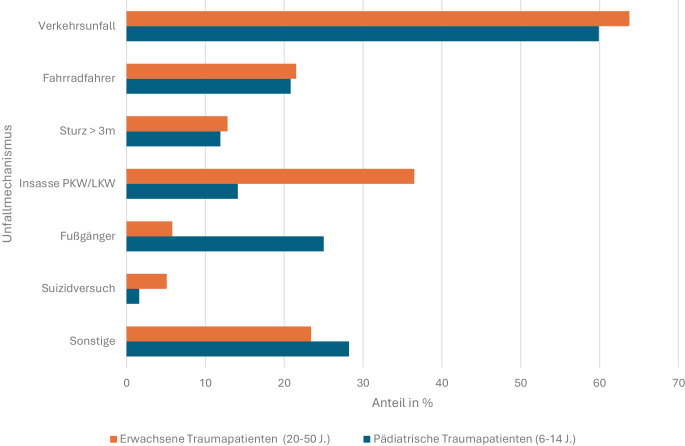
Unfallmechanismen bei Traumapatienten als Matched-Pair-Analyse aus Daten des Traumaregisters der DGU. Dargestellt sind die Anteile (in %) der jeweiligen Unfallmechanismen, getrennt nach pädiatrischen (6 bis 14 Jahre, *blau*) und erwachsenen (20 bis 50 Jahre, *orange*) Traumapatienten. Modifiziert nach Laurer et al. [42]

Wie bei Erwachsenen dominieren auch bei Kindern stumpfe Verletzungen [42]. In einer Matched-Pair-Analyse von 312 nach Verletzungsschwere gepaarten Fällen aus dem TraumaRegister DGU® zeigten Laurer et al., dass Kinder häufiger als Fußgänger, Erwachsene überwiegend als Fahrzeuginsassen verunglückten; suizidale Ereignisse waren bei Erwachsenen deutlich häufiger (5,1 % vs. 1,6 %) (Abb. 4; [42]). Die Verteilung der NACA-Scores war in mehreren Studien bei Kindern und Erwachsenen vergleichbar, ebenso der Anteil polytraumatisierter Patienten; Kinder wiesen jedoch häufiger isolierte Einzelverletzungen auf, insbesondere Schädel-Hirn-Traumata und thermische Traumen [28, 42]. Diese Unterschiede hatten keinen nachweisbaren Einfluss auf präklinische Therapie, Transportmittel oder Outcome [42].

## Diskussion

### Epidemiologie und klinische Besonderheiten

Schwer verletzte Kinder sind im Rettungsdienst selten. Sie weisen häufig komplexe Verletzungsmuster auf und sind durch altersabhängige anatomische und physiologische Besonderheiten und eine erschwerte klinische Beurteilbarkeit charakterisiert [53]. In vielen Studien nimmt die Inzidenz schwerer Verletzungen mit dem Alter zu; jüngere Kinder erleiden häufiger Schädel-Hirn- sowie Kopf‑/HWS-Verletzungen, während bei älteren Kindern und Jugendlichen eher Extremitäten- und Wirbelsäulenverletzungen dominieren [11, 27, 28, 64]. Verkehrsunfälle und Stürze stellen in den meisten untersuchten Kollektiven die häufigsten Traumamechanismen dar. Mehrere Arbeiten berichten zudem von einem Altersgipfel im Jugendalter sowie einer Überrepräsentation männlicher Patienten, die teilweise mit einer erhöhten traumabedingten Mortalität assoziiert ist [11, 27, 62, 64].

Für schwer verletzte Kinder zeigen nationale und internationale Studien eine Assoziation zwischen dem Einsatz von HEMS oder der primären Versorgung in überregionalen Traumazentren und einer geringeren Mortalität [5, 6, 47, 50]. Gleichzeitig verweisen andere Untersuchungen auf relevante Übertriageraten – sowohl bei Notarzteinsätzen als auch in Schockraumkohorten, in denen ein erheblicher Anteil der aufgenommenen Kinder noch am selben Tag wieder entlassen wird [21, 37].

### Strukturelle Herausforderungen und Notarztdichte

Mehrere Studien weisen darauf hin, dass invasive Maßnahmen bei pädiatrischen Patienten im präklinischen Setting, bezogen auf den einzelnen Notarzt, selten durchgeführt werden [15, 27, 48]. Vor diesem Hintergrund erscheinen zusätzliche klinische Erfahrung sowie zielgerichtetes Training notwendig, um Durchführungssicherheit aufbauen und erhalten zu können.

Gleichzeitig verändern gesundheitspolitische Entwicklungen die Rahmenbedingungen: Die angestrebte Zentralisierung der Notfallversorgung wird bislang nicht konsequent mit einer Bündelung pädiatrischer Expertise in definierten Zentren verknüpft. Schließungen kleinerer Kinderabteilungen können laut regionalen Analysen zu längeren Wegen, einer zunehmenden Belastung größerer Kliniken und zusätzlichen Sekundärverlegungen führen [29, 71], systematische Daten fehlen hierzu jedoch bislang. Im Gegensatz zu Ländern mit etablierten Traumanetzwerken und klar ausgewiesenen Paediatric Major Trauma Centres – wie etwa in Großbritannien [50] – besteht in Deutschland das Risiko regionaler Versorgungslücken [59].

Hinzu kommt eine im internationalen Vergleich außergewöhnlich hohe Dichte arztbesetzter Rettungsmittel. Kritisch kranke oder schwer verletzte Kinder verteilen sich dadurch auf viele ärztliche Teams (Deutschland 2 Notarztstützpunkte/100.000 Einwohner vs. Niederlande 0,025 Notarztstützpunkte/100.000 Einwohner). Auf Basis der Gesamtdaten der DRF Luftrettung über 5 Jahre berechnete Mockler, dass ein HEMS-Arzt im Mittel nur etwa alle 18 Monate ein kritisch krankes oder schwer verletztes Kind ≤ 10 Jahre versorgt, eine pädiatrische Intubation etwa alle 6 Jahre und eine Säuglingsintubation nur etwa alle 46 Jahre durchführt [48]. In stärker auf schwere Notfälle fokussierten HEMS-Systemen, wie in den Niederlanden, werden bei pädiatrischen Patienten höhere Interventionsraten, NACA-Scores und Mortalitätsraten bei Kindern berichtet [22, 56]: In einer retrospektiven Analyse niederländischer HEMS-Einsätze zeigten Gerritse et al. einen Anteil von 75 % schwerwiegend verletzter Kinder (NACA IV–VII) [22]. Zudem wurden bei 26,5 % der Patienten endotracheale Intubationen durchgeführt [22], und bei 9,2 % kam es zur Anlage intraossärer Zugänge [56].

Die neunte Stellungnahme der Regierungskommission zur Reform der Notfall- und Akutversorgung greift dieses Spannungsfeld auf und schlägt vor, die Befugnisse von Notfallsanitätern deutlich auszuweiten und spezialisierte Notärzte künftig v. a. bei besonders komplexen Einsätzen – überwiegend in der Luftrettung oder telemedizinisch – einzusetzen [9]. Für schwer verletzte Kinder würde dies bedeuten, seltene Hochrisikosituationen bei spezialisierten Teams zu bündeln, während die Mehrzahl pädiatrischer Notfälle protokollbasiert durch Notfallsanitäter versorgt werden könnte.

### Ausbildung, Training und Entscheidungsunterstützung

Mehrere Studien beschreiben Seltenheit, emotionale Belastung und eine uneinheitliche Ausbildung als Faktoren, die im Umgang mit kritisch verletzten Kindern zu Unsicherheiten und potenziell erhöhtem Fehlentscheidungsrisiko beitragen können [24, 26, 48, 55]. Zudem werden pädiatrische Einsätze von Notfallteams als besonders anspruchsvoll und häufig gefürchtet wahrgenommen [12, 24, 55]. Komplexe Maßnahmen wie Atemwegssicherung werden präklinisch selten durchgeführt, sodass praktische Fertigkeiten ohne ergänzende Trainingsangebote weder erlernt noch aufrechterhalten werden können [26, 49]. Die Daten von Mockler relativieren die Erwartung, dass zertifizierte Kindernotfallkurse diese Lücke allein schließen können: Es zeigte sich kein Zugewinn des globalen Sicherheitsempfindens bei Kindernotfällen, sondern nur Verbesserungen bei einzelnen Atemwegsmaßnahmen [49]. Demgegenüber war die Teilnahme an Erwachsenennotfallkursen mit einem höheren subjektiven Sicherheitsempfinden bei mehreren pädiatrischen Notfallbildern assoziiert, nicht jedoch beim schweren Schädel-Hirn-Trauma [49].

Gleichzeitig weisen Metaanalysen auf höhere Versagens- und Fehlpositionierungsraten der präklinischen Intubation bei Kindern im Vergleich zu Erwachsenen hin; zur sicheren Beherrschung sind zahlreiche supervidierte Intubationen erforderlich, klinische Gelegenheiten dafür werden jedoch seltener [14, 44, 45, 49, 60, 61]. Seltene, potenziell lebensrettende Prozeduren wie Thoraxdrainagen oder komplexere invasive Maßnahmen werden in Standard-Life-Support-Kursen meist nur randständig behandelt, denn die formalen pädiatrischen Mindestanforderungen an Notärzte sind gering [7, 49].

In der verfügbaren Literatur wird die Bedeutung kontinuierlicher, teamorientierter Trainingsformate sowie der strukturierten Umsetzung evidenzbasierter Algorithmen betont [27, 39, 48, 70]. Daher erscheint es plausibel, dass einmalige Kursbesuche ohne Auffrischung und lokale Implementierung nicht ausreichen, um eine nachhaltig hohe Versorgungsqualität herzustellen. Benötigt werden wiederkehrende, zielgruppenorientierte Simulationstrainings in regionalen Netzwerken, in denen technische Fertigkeiten und Non-Technical Skills eingeübt werden. Sie können fundierte klinische Erfahrung jedoch nur ergänzen, nicht ersetzen; die sichere Versorgung schwer verletzter und kritisch erkrankter Kinder bleibt auf praxisnahe klinische Erfahrung angewiesen [48]. Eine standardisierte pädiatrische Ausrüstung mit altersadaptierten Dosierungshilfen und klar strukturierten Materialsets kann Entscheidungen in Stresssituationen zusätzlich erleichtern [26, 33, 34].

Neben Ausbildung und Strukturmaßnahmen bietet die Telemedizin zusätzliche Chancen. Deutsche und internationale Modelle regionaler Kindernotarztsysteme und spezialisierter pädiatrischer Retrieval-Netzwerke zeigen, dass hochspezialisierte Teams den Rettungsdienst vor Ort effektiv unterstützen können [32, 35]. Ein ausgebautes, auch pädiatrisches Telenotarztsystem könnte Entscheidungsprozesse insbesondere in dünn besiedelten Regionen und in seltenen Hochrisikosituationen unterstützen. Fehlermanagementsysteme (z. B. CIRS), Einsatznachbesprechungen und psychologische Unterstützung der Einsatzkräfte sind weitere Bausteine zur Erhöhung der Patientensicherheit und zur Stärkung der Resilienz [24, 26].

Ein besonderer Aspekt ist die Erkennung nichtakzidentieller Verletzungen; hier sind spezifische Schulungen, standardisierte Screeninginstrumente und klar definierte Meldewege essenziell [4].

### Datenlage, Registerstrukturen und internationale Perspektive

Ein zentrales Hindernis für Qualitätsentwicklung ist die begrenzte und heterogene Datenlage. Einsatzdokumentationen sind häufig unvollständig oder retrospektiv fehleranfällig [36, 69], und eine systematische Verknüpfung präklinischer Daten mit klinischen Outcomes ist in Deutschland bisher nur in Ausnahmen möglich [48]. Damit bleiben zentrale Fragen zur Ergebnisqualität der präklinischen Kindertraumaversorgung unbeantwortet.

Eine aktuelle Auswertung von Eimer et al. zeigt, dass in der deutschen Luftrettung bei pädiatrischen Traumapatienten grundsätzlich eine wirksame präklinische Analgesie erreicht wird [16]; dieser Aspekt wurde in der vorliegenden Arbeit nicht weiter vertieft, da die detaillierte Bewertung der Schmerztherapie nicht im Fokus der Fragestellung lag.

Länder mit etablierten Traumanetzwerken und verknüpfbaren Registern – etwa Großbritannien (NMTR) oder Skandinavien – zeigen, dass Outcome-basierte Evaluationen und Register die Grundlage für erfolgreiche Strukturreformen bilden können [50–52, 54]. Die epidemiologischen Muster pädiatrischer Traumata sind dabei in Hochlohnländern insgesamt ähnlich: überwiegend stumpfe, unbeabsichtigte Verletzungen, ein Altersgipfel im Jugendalter und eine Dominanz männlicher Patienten [11, 22, 40, 52, 62]. Unterschiede ergeben sich v. a. in den Versorgungsstrukturen: Viele Länder arbeiten mit Paramedic-basierten Systemen und wenigen spezialisierten ärztlichen Teams [13, 40, 51, 54], während Deutschland durch eine hohe Notarztdichte bei zugleich fragmentierten Netzwerkstrukturen gekennzeichnet ist [23, 32].

In Low-Income- und Middle-Income-Ländern ist die Traumamortalität im Kindesalter deutlich höher; präklinische Systeme sind häufig nur rudimentär vorhanden [57]. Die hier dargestellte Diskussion spiegelt damit v. a. die Herausforderungen eines hochentwickelten Systems wider. Konzepte wie standardisierte prä- und innerklinische Datenerfassung, Registerverknüpfung, regionalisierte Versorgungsnetzwerke und strukturiertes Training des präklinischen Personals können als Modelle für die Weiterentwicklung der Kindertraumaversorgung dienen.

## Limitationen

Diese Arbeit ist eine narrative, nichtsystematische Übersichtsarbeit. Die Literaturauswahl beruht auf PubMed-Suche und Rückwärtssuche und kann durch Publikations- und Selektionsbias verzerrt sein. Die eingeschlossenen Studien unterscheiden sich deutlich in Design, Einschlusskriterien, Altersgrenzen und Versorgungsstrukturen; quantitative Vergleiche sind daher nur eingeschränkt möglich.

Obwohl Eimer et al. nationale Daten zur präklinischen Analgesie bei Kindern beitrugen, bleibt die deutsche Evidenzlage zu diesem Thema insgesamt dünn. Internationale Studien sind aufgrund unterschiedlicher Versorgungsstrukturen nur begrenzt übertragbar.

Daten zur Verletzungsschwere sind nur begrenzt aussagekräftig, da in den Quellen nicht immer zwischen erkrankten und verletzten Kindern oder zwischen Luft- und Bodenrettung unterschieden wurde; zudem variieren die eingeschlossenen Kollektive stark, etwa hinsichtlich Altersgrenzen oder der Einbeziehung nur schwer verletzter vs. aller verletzten Kinder.

Ein Großteil der deutschen Daten stammt aus Register- und Beobachtungsstudien über einen Zeitraum von mehr als 20 Jahren. In dieser Zeit haben sich Rettungsdienststrukturen, klinische Standards und Verkehrssicherheit wesentlich verändert, sodass ältere Kollektive nur bedingt auf die aktuelle Versorgungssituation übertragbar sind.

Internationale Daten sind nur bedingt auf Deutschland übertragbar, weil viele Studien aus Paramedic-basierten, teils stärker zentralisierten Rettungssystemen stammen. Patientenpopulation, Alarmierungs- und Dispositionsstrategien sowie die rechtlichen Rahmenbedingungen unterscheiden sich dabei teils deutlich.

Zudem liegt ein Schwerpunkt dieser Übersichtsarbeit auf deutschsprachigen und, durch die eingeschränkte Datenlage bedingt, HEMS-Systemen; andere Präklinikmodelle werden nur exemplarisch berücksichtigt. Die Schlussfolgerungen sind daher v. a. als Impuls für weitergehende, prospektive und registerbasierte Untersuchungen und zur kritischen Reflexion eigener Strukturen zu verstehen.

## Fazit für die Praxis und Rückbezug auf das ursprüngliche Notarztkonzept

Ursprünglich war der arztbesetzte Rettungsdienst in Deutschland als hochspezialisierte Ressource für komplexe Notfälle konzipiert. In Göttingen wurde der 1970 eingeführte Notarztwagen von Stoffregen als „verlängerter Arm der Intensivstation“ beschrieben; bereits am zweiten Einsatztag konnte er bei einem 4‑jährigen Kind nach einem Ertrinkungsunfall lebensrettend eingesetzt werden [74].

Dieses Verständnis – Notärzte als intensivmedizinisch versierte Spezialressource für wenige, hochkomplexe Fälle – steht im Kontrast zur heutigen, flächendeckenden Notarztdichte mit geringer Exposition in der Versorgung schwer verletzter Kinder.

Die zusammengetragenen Daten sprechen dafür, das ursprüngliche Konzept im Sinne der Regierungskommission wieder stärker aufzugreifen: Schwer verletzte Kinder sollten als prototypische Patientengruppe verstanden werden, bei der der Notarztdienst tatsächlich als „verlängerter Arm der Intensivstation“ fungiert – mit versierten Spezialteams in Schlüsselrollen und einem qualifizierten, notfallsanitäterbasierten System für die häufigeren, weniger schweren pädiatrischen Notfälle. Voraussetzungen sind klare Netzwerkstrukturen, definierte Kompetenzprofile, wiederkehrende hochwertige Trainingsangebote und eine verbesserte Datenbasis, die eine kontinuierliche Evaluation und Weiterentwicklung der präklinischen Kindertraumaversorgung ermöglicht.

## Abkürzungen

CIRS: Critical Incident Reporting System
GCS: Glasgow Coma Scale
GEMS: Ground Emergency Medical Services
HEMS: Helicopter Emergency Medical Services
i.o.: Intraossär
ISS: Injury Severity Score
i.v.: Intravenös
HWS: Halswirbelsäule
NACA: National Advisory Committee for Aeronautics
NMTR: National Major Trauma Registry
NTDB: National Trauma Data Bank
PKW: Personenkraftwagen
SHT: Schädel-Hirn-Trauma
TR-DGU®: TraumaRegister DGU® der Deutschen Gesellschaft für Unfallchirurgie
